# Supplementary material for: Heat adaptation of phage T7 under an extended genetic code
Source: Virus Evol. 2021 Dec 1;7(2):veab100. doi: 10.1093/ve/veab100 (PMC8923235; doi:10.1093/ve/veab100)
Supplement: veab100_Supp [file veab100_supp.zip › Sequencing_Analysis.zip!/Sequencing_Analysis/A1/evidence/JC_SIDE_1_54.html]

BRESEQ :: Evidence


| New junction evidence | | | | | | | | | | | |
| --- | --- | --- | --- | --- | --- | --- | --- | --- | --- | --- | --- |
|  | | seq id | position | reads (cov) | reads (cov) | score | skew | freq | annotation | gene | product |
| \* | ? | NC\_001604.1 | = 10002 | 2433 (0.790) | 15 (0.000) | 14/594 | 38.4 | 0.51% | intergenic (–/–) | *–/–* | –/– |
|  | ? | NC\_001604.1 | 10186 = | 3422 (1.120) |  |  |  |  | intergenic (–/–) | *–/–* | –/– |
| Rejected: Coverage evenness skew score above cutoff. | | | | | | | | | | | |
| Rejected: Frequency below/above cutoff threshold. | | | | | | | | | | | |

Only 100 of 2453 total aligned reads displayed.  

`CCTTTGGTGGCGGTGAAGACGATTGGGCTGACGAAGTTGAAGAGAACGGCTATGTTGCCTCTGGTTCTGCCAAAGCGAGCAAACCACGCGACGAAGAAAGCTGGGACGAAGACGACGAAGAGTCCGAGGAAGCAGACGAAGACGGAGACTTCTAAGTGGAACTGCGGGAGAAAATCCTTGAGCGAATCAAGGTGACTTCCTCTGGGTGTTGGGAGTGGCAGGGCGCTACGAACAATAAAGGGTACGGGCAGGTGTGGTGCAGCAATACCGGAAAGGTTGTCTACTGTCATCGCGTAATGTCTAATGCTCCGAAAGGTTCTACCGTCCTGCACTCCTGTGATAATCCATTATGTTGTAACCCTGAACACCTATCCATAGGAACTCCAAAAGAGAACTCCACTGACATGGTAAATAAGGGTCGCTCACACAAGGGGTATAAACTTTCAGACGAAGACGTAATGGCAATCATGGAGTCCAGCGAGTCCAATGTATCCTTAGCTCGCACCTATGG‑TGTCTCCCAACAGACTATTTGTGATATACGCAAAGGGAGGCGACATGGCAGGTTACGGCGCTAAAGGAATCCGAAAGGTTGGAGCGTTTC  >  NC_001604.1/9702‑10302`  

                                                                                                                                                                                                                                                                                                            `|`                                                                                                                                                                                                                                                                                                               
`ccTTTGGTGGCGGTGAAGACGATTGGGCTGACGAAGTTGAAGAGAACGGCTATGTTGCCTCTGGTTCTGCCAAAGTGAGCAAACCACGCGACGAAGAAAGCTGGGACGAAGACGACGAAGAGTCCGAGGAAGCAGACGAAGACGGAGACTTCTAAGTGGAACTGCGGGAGAAAATCCTTGAGCGAATCAAGGTGACTTCCTCTGGGTGTTGGGAGTGGCAGGGCGCTACGAACAATAAAGGGTACGGGCAGGTGTGGTGCAGCAATACCGGAAAGGTTGTCTACTGTCATCGCGTAATGTc                                                                                                                                                                                                                                                                                                               >  1:159357/1‑301 (MQ=255)`  
`ccTTTGGTGGCGGTGAAGACGATTGGGCTGACGAAGTTGAAGAGAACGGCTATGTTGCCTCTGGTTCTGCCAAAGCGAGCAAACCACGCGACGAAGAAAGCTGGGACGAAGACGACGAAGAGTCCGAGGAAGCAGACGAAGACGGAGACTTCTAAGTGGAACTGCGGGAGAAAATCCTTGAGCGAATCAAGGTGACTTCCTCTGGGTGTTGGGAGTGGCAGGGCGCTACGAACAATAAAGGGTACGGGCAGGTGTGGTGCAGCAATACCGGAAAGGTTGTCTACTGTCATCGCGTAATGTc                                                                                                                                                                                                                                                                                                               >  1:141598/1‑301 (MQ=255)`  
            `gTGAAGACGATTGGGCTGACGAAGTTGAAGAGAACGGCTATGTTGCCTCTGGTTCTGCCAAAGCGAGCAAACCACGCGACGAAGAAAGCTGGGACGAAGACGACGAAGAGTCCGAGGAAGCAGACGAAGACGGAGACTTCTAAGTGGAACTGCGGGAGAAAATCCTTGAGCGAATCAAGGTGACTTCCTCTGGGTGTTGGGAGTGGCAGGGCGCTACGAACAATAAAGGGTACGGGCAGGTGTGGTGCAGCAATACCGGAAAGGTTGTCTACTGTCATCGCGTAATGTCTAATGCTCCGaa                                                                                                                                                                                                                                                                                                   >  1:404758/1‑301 (MQ=255)`  
             `ggAAGACGATTGGGCTGACGAAGTTGAAGAGAACGGCTATGTAGCCTCTGGTTCTGCCAAATCGAGCAAACCACGCTACGAAGAAAGCTGGGGCGGAGACGACGAAGAGTCCGAGGAAGCAGACGAAGACGGAGACTTCTAAGTGGAACTGCGGGAGAAAATCCTTGATCGAATCAAGGTGACTTCCTCTGGGTGTTGGGAGTGGCAGGGCGCTACGAACAATAAAGGGTACGGGCAGGTGTGGTGCAGCAATACCGGAAAGGTTGTCTACTGTCATCGCGTAATGTCTAATGCTCCGaaa                                                                                                                                                                                                                                                                                                  <  1:175717/300‑1 (MQ=255)`  
              `gAAGACGATTGGGCTGACGAAGTTGAAGAGAACGGCTATGTTGCCTCTGGTTCTGCCAAAGCGAGCAAACCACGCGACGAAGAAAGCTGGGACGAAGACGACGAAGAGTCCGAGGAAGCAGACGAAGACGGAGACTTCTAAGTGGAACTGCGGGAGAAAATCCTTGAGCGAATCAAGGTGACTTCCTCTGGGTGTTAGGAGTGGCAGGGCGCTACGAACAATAAAGGGTACGGGCAGGTGTGGTGCAGCAATACCGGAAAGGTTGTCTACTGTCATCGCGTAATGTCTAATGcctgtctct                                                                                                                                                                                                                                                                                                 >  1:204739/1‑293 (MQ=255)`  
                `aGACGATTGGGCTGACGAAGTTGAAGAGAACGGCTATGTTGCCTCTGGTTCTGCCACAGCGAGCAAACCACGCGACGAAGAAAGCTGGGACGAAGACGACGAAGAGTCCGAGGAAGCAGACGAAGACGGAGACTTCTAAGTGGAACTGCGGGAGAAAATCCTTGAGCGAATCAAGGTGACTTCATCTGGGTGTTGGGAGTGGCAGGGCGCTACGAACAATAAAGGGTACGGGCAGGTGTGGTGCAGCAATACCGGCCAGGTTGTCTACTGTCATCGCGTCATGTCTACTGcatgtctctta                                                                                                                                                                                                                                                                                               >  1:279524/1‑291 (MQ=255)`  
                `aGACGATTGGGCTGACGAAGTTGAAGAGAACGGCTATGTTGCCTCTGGTTCTGCCAAAGCGAGCAAACCACGCGACGAAGAAAGCTGGGACGAAGACGACGAAGAGTCCGAGGAAGCAGACGAAGACGGAGACTTCTAAGTGGAACTGCGGGAGAAAATCCTTGAGCGAATCAAGGTGACTTCCTCTGGGTGTTGGGAGTGGCAGGGCGCTACGAACAATAAAGGGTACGGGCAGGTGAGGTGCAGCAATACCGGAAAGGTTGTCTACTGTCATCGCGTAATGTCTAATGCTCCGAAAGGt                                                                                                                                                                                                                                                                                               >  1:117937/1‑301 (MQ=255)`  
                   `cGATTGGGCTGACGAAGTTGAAGAGAACGTCTATGTTGCCTCTGATTCTGCCAAAGCGAGCAAACCACGCGACGAAGAAAGCTGGGACGAAGACGACGAAGAGTCCGAGGAAGCAGACGAAGACGGAGACTTCTAAGTGGAACTGCGGGAGAAAATACTTGAGCGAATCAAGGTGCCTTCCTCTGGGTGTTGGGAGTGGCAGGTCGCTACGAACAATAAAGGGTACGGGCAGGGGTGGTGCAGCAATACCGAACAGGTTGTCTACTGTCATCGCGTAATGTCTAATGCTACGAAAGGTTCt                                                                                                                                                                                                                                                                                            >  1:17252/1‑301 (MQ=255)`  
                   `cGATTGGGCTGACGAAGTTGAAGAGAACGGCTATGTTGCCTCTGGTTCTGCCAAAGCGAGCAAACCACGCGACGAAGAAAGCTGGGACGAAGACGACGAAGAGTCCGAGGAAGCAGACGAAGACGGAGACTTCTAAGTGGAACTGCGGGAGAAAATCCTTGAGCGAATCAAGGTGACTTCCTCTGGGTGTTGGGAGTGGCAGGGCGCTACGAACAATAAAGGGTACGGGCAGGTGTGGTGCAGCAATACCGGAAAGGTTGTCTACTGTCATCGCGTAATGTCTAATGCTACGAAAGGTTCt                                                                                                                                                                                                                                                                                            >  1:154464/1‑301 (MQ=255)`  
                        `gggCTGACGAAGTTGAAGAGAACGGCTATGTTGCCTCTGGTTCTGCCAAAGCGAGCAAACCACGCGACGAAGAAAGCTGGGACGAAGACGACGAAGAGTCCGAGGAAGCAGACGAAGACGGAGACTTCTAAGTGGAACTGCGGGAGAAAATCCTTGAGCGAATCAAGGTGACTTCCTCTGGGTGTTGGGAGTGGCAGGGCGCTACGAACAATAAAGGGTACGGGCAGGTGTGGTGCAGCAATACCGGAAAGGTTGTCTACTGTCATCGCGTAATGTCTAATGCTCCGAAAGGTTCTACCGt                                                                                                                                                                                                                                                                                       >  1:470630/1‑301 (MQ=255)`  
                               `cGAAGTTGAAGAGAACGGCTATGTTGCCTCGGGTTCTGCCAAAGCGAGCAAACCACGCGACGAAGAAAGCTGGGACGAAGACGACGAAGAGTCCGAGGAAGCAGACGAAGACGGAGACTTCTAAGTGGAACTGCGGGAGAAAATCCTTGAGCGAATCAAGGTGACTTCCTCTGGGTGTTGGGAGTGGCAGGGCGCTACGAACAATAAAGGGTACGGGCAGGTGTGGTGCAGCAATACCGGAAAGGTTGTCTACTGTCATCGCGTAATGTCTAATGCTCCGAAAGGTTCTACCGTCCTGCAc                                                                                                                                                                                                                                                                                <  1:440322/301‑1 (MQ=255)`  
                                 `aaGTTGAAGAGAACGGCTATGTTGCCTCTGGTTCTGCCAAAGCGAGCAAACCACGCGACGAAGAAAGCTGGGACGAAGACGACGAAGAGTCCGAGGAAGCAGACGAAGACGGAGACTTCTAAGTGGAACTGCGGGAGAAAATCCTTGAGCGAATCCAGGTGACTTCCTCTGGGTGTTGGGAGTGGCAGGGCGCTACGAACAATAAAGGGTACGGGCAGGTGTGGTGCAGCAATACCGGAAAGGTTATCTACTGTCATCGCGTAATGTATAATGCTACGAAAGGTTCTACCGTCCTGCAcgc                                                                                                                                                                                                                                                                              >  1:248847/1‑299 (MQ=255)`  
                                  `aGTTGAAGAGAACTGCTATGTTGCCTCTGGTTCTGCCAAAGCGAGCAAACCACGCGACGAAGAAAGCTGGGACGAAGACGACGAAGAGTCCGAGGAAGCAGACGAAGACGGAGACTTCTAAGTGGAACTGCGGGAGAAAATCCTTGAGCGAATCAAGGTGACTTCCTCTGGGTGTTGGGAGTGGCAGGGCGCTACGAACAATAAAGGGTACGGGCAGGTGTGGTGCAGCAATACCGGAAAGGTTGTCTACTGTCATCGCGTAATGTCTAATGCTCCGAAAGGTTCTACCGTCCTGCACTcc                                                                                                                                                                                                                                                                             >  1:480839/1‑301 (MQ=255)`  
                                  `aGTTGAAGAGAACGGCTATGTTGCCTCTGGTTCTGCCAAAGCGAGCAAACCACGCGACGAAGAAAGCTGGGACGAAGACGACGAAGAGTCCGAGGAAGCAGACGAAGACGGAGACTTCTAAGTGGAACTGCGGGAGAAAATCCTTGAGCGAATCAAGGTTACTTCCTCTGGGTGTTGGGAGTGGCAGGGCGCTACGAACAATAAAGGGTACGGGCAGGTGTGGTGCAGCAATACCGGAAAGGTTGTCTACTGTCATCGCGTAATGTCTAATGCTCCGAAAGGTTCTACCGTCCTGCACTcc                                                                                                                                                                                                                                                                             >  1:498975/1‑301 (MQ=255)`  
                                   `gTTGAAGAGAACGGCTATGTTGCCTCTGGTTCTGCCAAAGCGAGCAAACCACGCGACGAAGAAAGCTGGGACGAAGACGACGAAGAGTCCGAGGAAGCAGACGAAGACGGAGACTTCTAAGTGGAACTGCGGGAGAAAATCCTTGAGCGAATCAAGGTGACTTCCTCTGGGTGTTGTGAGTGGCAGGGCGCTACGAACAATAAAGGGTACGGGCAGGTGTGGTGCAGCAATACCGGAAAGGTTGTCTACTGTCATCGCGTAATGTCTAATGCTCCGAAAGGTTCTACCGTCCTGCACTCCt                                                                                                                                                                                                                                                                            >  1:41010/1‑301 (MQ=255)`  
                                   `gTTGAAGAGAACGGCTATGTTGCCTCTGGTTCTGCCAAAGCGAGCAAACCACGCGACGAAGAAAGCTGGGACGAAGACGACGAAGAGTCCGAGGAAGCAGACGAAGACGGAGACTTCTAAGTGGAACTGCGGGAGAAAATCCTTGAGCGAATCAAGGTGACTTCCTCTGGGTGTTGGGAGTGGCAGGGCGCTACGAACAATAAAGGGTACGGGCAGGTGTGGTGCAGCAATACCGGAAAGGTTGTCTACTGTCATCGAGTAATGTCTAATGCTCCGAAAGGTTCTACCGTctgtctcttat                                                                                                                                                                                                                                                                            >  1:430697/1‑291 (MQ=255)`  
                                   `gTTGAAGAGAACGGCTATGTTGCCTCTGGTTCTGCCAAAGCGAGCAAACCACGCGACGAAGAAAGCTGGGACGAAGACGACGAAGAGTCCGAGGAAGCAGACGAAGACGGAGACTTCTAAGTGGAACTGCGGGAGAAAATCCTTGAGCGAATCAAGGTGACTTCCTCTGGGTGTTGGGAGTGGCAGGGCGCTACGAACAATAAAGGGTACGGGAAGGTGTGGTGCAGCAATACCGGAAAGGTTGTCTACTGTCATCGCGTAATGTCTAATGCTCCGAAAGGtctgtctcttatacacattt                                                                                                                                                                                                                                                                            >  1:323172/1‑282 (MQ=255)`  
                                   `gTTGAAGAGAACGGCTATGTTGCCTCTGGTTCTGCCAAAGCGAGCAAACCACGCGACGAAGAAAGCTGGGACGAAGACGACGAAGAGTCCGAGGAAGCAGACGAAGACGGAGACTTCTAAGTGGAACTGCGGGAGAAAATCCTTGAGCGAATCAAGGTGACTTCCTCTGGGTGTTGGGAGTGGAAGGGCGCTACGAACAATAAAGGGTACGGGCAGGTGTGGTGCAGCAATACCGGAAAGGTTGTCTACTGTCATCGCGTAATGTCTAATGCTCCGAAAGGTTCTACCGTCCTGCACTccc                                                                                                                                                                                                                                                                            >  1:259533/1‑300 (MQ=255)`  
                                      `gAAGAGAACGGCTATGTTGCCTCTGGTTCTGCCAAAGCGAGCAAACCACGCGACGAAGAAAGCTGGGACGAAGACGACGAAGAGTCCGAGGAAGCAGACGAAGACGGAGACTTCTAAGTGGAACTGCGGGAGAAAATCCTTGAGCGAATCAAGGTGACTTCCTCTGGGTGTTGGGAGTGGCAGGGCGCTACGAACAATAAAGGGTACGGGCAGGTGTGGTGCAGCAATACCGGAAAGGTTGTCTACTGTCATCGCGTAATGTCTAATGCGCCGAAAGGTTCTACCGTCCTGCAcctgtctc                                                                                                                                                                                                                                                                         >  1:225152/1‑294 (MQ=255)`  
                                        `agagAACGGCTATGTTGCCTCTGGTTCTGCCAAAGCGAGCAAACCACGCGACGAAGAAAGCTGGGACGAAGACGACGAAGAGTCCGAGGAAGCAGACGAAGACGGAGACTTCTAAGTGGAACTGCGGGAGAAAATCCTTGAGCGAATCAAGGTGACTTCCTCTGGGTGTTGGGAGTGGCAGGGCGCTACGAACAATAAAGGGTACGGGCAGGTGTGGTGCAGCAATACCGGAAAGGTTGTCTACTGTCATCGCGTAATGTCTAATGCTCCGAAAGGTTCTACCGTCCTGCACTCCTGTGAt                                                                                                                                                                                                                                                                       <  1:12137/301‑1 (MQ=255)`  
                                        `agagAACGGCTAGGTTGCCTCTGGTTCTGCCAAAGCGAGAAAACCACGCGACGAAGAAAGCTGGGGCGAAGACGACGAAGAGTCCGAGGAAGCAGACGAAGACGGAGACTTCTAAGTGGAACTGCGGGAGAAAATCCTTGAGCGAATCAAGGTGACTTCCTCTGGGTGTTGGGAGTGGCAGGGCGCTACGAACAATAAAGGGTACGGGCAGGTGTGGTGCAGCAATACCGGAAAGGTTGTCTACTGTCATCGCGTAATGTCTAATGCTCAGAAAGGTTCTACCGTCCTGCACTCCTGTGAt                                                                                                                                                                                                                                                                       <  1:229441/301‑1 (MQ=255)`  
                                           `gaACGGCTATGTTGCCTCTGGTTCTGCCAAAGCGAGCAAACCACGCGACGAAGAAAGCTGGGACGAAGACGACGAAGAGTCCGAGGAAGCAGACGAAGACGGAGACTTCTAAGTGGAACTGCGGGAGAAAATCCTTGAGCGAATCAAGGTGACTTCCTCTGGGTGTTGGGAGTGGCAGGGCGCCACGAACAATAAAGGGTACTGGCAGGTGTGGTGCAGCAATACCGGAAATGTTGTCTACTGTCATCGCGTAATGTCTAATGCTCCGAAAGGCTCTACCGTCCTGCAcctgtctcttatt                                                                                                                                                                                                                                                                    >  1:351130/1‑289 (MQ=255)`  
                                           `gaACGGCTATGTTGCCTCTGGTTCTGCCAAAGCGAGCAAACCACGCGACGAAGAAAGCTGGGACGAAGACGACGAAGAGTCCGAGGAAGCAGACGAAGACGGAGACTTCTAAGTGGAACTGAGGGAGAAAATCCTTGAGCGAATCAAGGTGACTTCCTCTGGGTGTTGGGAGTGGCAGGGCGCTACGAACAATAAAGGGTACGGGCAGGTGTGGTGCAGCAATACCGGAAAGGTTGTCTACTGTCATCGCGTAATGTCTAATGCTCCGAAAGGTTCTACCGTccctgtctcttatacacat                                                                                                                                                                                                                                                                    >  1:208460/1‑284 (MQ=255)`  
                                                 `cTATGTTGCCTCTGGTTCTGCCAAAGCGAGCAAACCACGCGACGAAGAAAGCTGGGACGAAGACGACGAAGAGTCCGAGGAAGCAGACGAAGACGGAGACTTCTAAGTGGAACTGCGGGAGAAAATCCTTGAGCGAATCAAGGTGACTTCCTCTGGGTGTTGGGAGTGGCAGGGCGCTACGAACAATAAAGGGTACGGGCAGGTGTGGTGCAGCAATACCGGAAAGGTTGTCTACTGTCATCGCGTAATGTCTAATGCTCCGAAAGGTTCTACCGTCCTGCACTCCTGTGATAATCCATTa                                                                                                                                                                                                                                                              >  1:424625/1‑301 (MQ=255)`  
                                                      `ttGCCTCTGGTTCTGCCAAAGCGAGCAAACCACGCGACGAAGAAAGCTGGGACGAAGACGACGAAGAGTCCGAGGAAGCAGACGAAGACGGAGACTTCTAAGTGGAACTGCGGGAGAAAATCCTTGAGCGAATCAAGGTGACTTCCTCTGGGTGTTGGGAGTGGCAGGGCGCTACGAACAATAAAGGGTACGGGCAGGTGTGGTGCAGCAATACCGGAAAGGTTGTCTACTGTCATCGCGTAATGTCTAATGCTCCGAAAGGTTCTACCGTCCTGCACTCCTGTGATAATCCATTAtgttg                                                                                                                                                                                                                                                         <  1:381205/301‑1 (MQ=255)`  
                                                        `gCCTCTGGTTCTGCCAAAGCGAGCAAACCACGCGACGAAGAAAGCTGGGACGAAGACGACGAAGAGTCCGAGGAAGCAGACGAAGACGGATACTTCTAAGTGGACCTGCGGGAGAAAATCCTTGAGCGAATCAAGGTGACTTCCTCGGGGTGTTGGGATTGGCAGGGCGCTACTAACAATAAACGGTACGGGCAGGTGTGGTTCAGCCATACCGGAAAGGTTTTCTACTGTCATCGCGTAATTTCTAATGCTCCGACATGTTGTACCTTCCTGCCCTCCTGTGATATTCCCTCAtgttgtt                                                                                                                                                                                                                                                       >  1:508963/1‑300 (MQ=255)`  
                                                          `ctctGGTTCTGCCAAAGCGAGCAAACCACGCGACGAAGAAAGCTGGGACGAAGACGACGAAGAGTCCGAGGAAGCAGACGAAGACGGAGACTTCTAAGTGGAACTGCGGGAGTAAATCCTTGAGCGAATCAAGGTGACTTCCTCTGGGTGTTGGGAGTGGCAGGGCGCTACGAACAATAAAGGGTACGGGCAGGTGTGGTGCAGCAATACCGGAAAGGTTGTCTACTGTCATCGCGTAATGTCTAATGCTCCGAAAGGTTCTACCGTCCTGCACTCCTGTGATAATCACTTCtgtttgaac                                                                                                                                                                                                                                                     >  1:54767/1‑296 (MQ=255)`  
                                                           `tctGGTTCTGGCAAAGCGGGCAAAACACGCGACGAAGAAAGCGGGGACGAAAACGACGAAGAGTCAGAGGAAGCAGACGAAGACGGAGACTTCGAAGTGGAACTGCGGGAGAAAATCCTTGAGCGAATCAAGGTGACTTCCTCTGGGTGTTGGGAGTGGCAGGGCGCTACGAACAATAAAGGGTACGGGCAGGTGTGGTGCAGCAATACCGGAAAGGTTGTCTACTGTCATCGCGTAATGTCTAATGCTCCGAAAGGTTCTACCGTCCTGCACTCCTGTGATAATCCATTATGTTGTAAcc                                                                                                                                                                                                                                                    <  1:485425/301‑1 (MQ=255)`  
                                                              `ggTTCTGCCAAAGCGAGCAAACCACGCGACGAAGAAAGCTGGGACGAAGACGACGAAGAGTCCGAGGAAGCAGACGAAGACGGAGACTTCTAAGTGGAACTGCGGGAGAAAATCCTTTAGCGAATCAAGGTGACTTCCTCTGGGTGTTGGGAGTGGCAGGGCGCTACGAACAATAAAGGGTACGGGCAGGTGTGGTGCAGCAATACCGGAAAGGTTGTCTACTGTCATCGCGTAATGTCTAATGCTCCGAAAGGTTCTACCGTCCTGCACTCCTGTGATAATCCATTATGTTGTAACCCTg                                                                                                                                                                                                                                                 >  1:137045/1‑301 (MQ=255)`  
                                                               `gTTCTGCCAAATCGAGCAAACCACGCGACGAAGAAAGCTGGGACGAAGACGACGAAGAGTCCGAGGAAGCAGACGAAGACGGAGACTTCTAAGTGGAACTGCGGGAGAAAATCCTTGAGCGAATCAAGGTGACTTCCTCTGGGTGTTGGGAGTGGCAGGGCGCTACGAACAATAAAGGGTACGGGCAGGTGTGGTGCAGCAATACCGGAAAGGTTGTCTACTGTCATCGCGTAATGTCTAATGCTCCGAAAGGTTCTACCGTCCTGCACTCCTGTGATAATCCATTAtgtctgtctcttat                                                                                                                                                                                                                                                >  1:63012/1‑290 (MQ=255)`  
                                                                   `tGCCAAAGCGAGCAAACCACGCGACGAAGAAAGCTGGGACGAAGACGACGAAGAGTCCGAGGAAGCAGACGAAGACGGAGACTTCTAAGTGGAACTGCGGGAGAAAATCCTTGAGCGAATCAAGGTGACTTCCTCTGGGTGTTGGGAGTGGCAGGGCGCTACGAACAATAAAGGGTACGGGCAGGAGTGGTGCAGCAATACCGGAAAGGTTGTCTACTGTCATCGCGTAATGTCTAATGCTCCGAAAGGTTCTACCGTCCTGCACTCCTGTGATAATCCATTATGTTGTAACCCTGAacac                                                                                                                                                                                                                                            <  1:180372/301‑1 (MQ=255)`  
                                                                   `tGCCAAAGCGAGCAAACCACGCCACGAAGAAAGCTGGGACGAAGACGACGAAGAGTCCGAGGAAGCAGACGAAGACGGAGACTTCTAAGTGGAACTGCGGGAGAAAATCCTTGAGCGAATCAAGGTGACTTCCTCTGGGTGTTGGGAGTGGCAGGGCGCTACGAACAATAAAGGGTACGGGCAGGTGTGGTGCAGCAATACCGGAAAGGTTGTCTACTGTTATCGCGTAATGTCTAATGCTCCGAAAGGTTCTACCGTCCTGCACTCCTGTGATAATCCATTATGTTGTAACCCTGAacac                                                                                                                                                                                                                                            <  1:163356/301‑1 (MQ=255)`  
                                                                    `gCCAAAGCGAGCAAACCACGCGACGAAGAAAGCTGGGACGAAGACGACGAAGAGTCCGAGGAAGCAGACGAAGACGGAGACTTCTAAGTGGAACTGCGGGAGAAAATCCTTGAGCGAATCAAGGTGACTTCCTCTGGGTGTTGGGAGTGGCAGGGCGCTACGAACAATAAAGGGTACGGGCAGGTGTGGTGCAGCAATACCGGAAAGGTTGTCTACTGTCATCGCGTAATGTCTAATGCTCCGAAAGGTTCTACCGTCCTGCACTCCTGTGAtctgtctcttatacacatctccgagccca                                                                                                                                                                                                                                           >  1:41442/1‑273 (MQ=255)`  
                                                                    `gCCAAAGCGAGCAAACCACGCGACGAAGAAAGCTGGGACGAAGACGACGAAGAGTCCGAGGAAGCAGACGAAGACGGAGACTTCTAAGTGGAACTGCGGGAGAAAATCCTTGAGCGAATCAAGGTGACTTCCTCTGGGTGTTGGGAGTGGCAGGGCGCTACGAACAATAAAGGGTACGGGCAGGTGTGGTGCAGCAATACCGGAAAGGTTGTCTACTGTCATCGCGTAATGTCTAATGCTCCGAAAGGTTCTACCGTCCTGCACTCCTGTGATAATCCATTATGTTGTAACCCTGAACAcc                                                                                                                                                                                                                                           >  1:25348/1‑301 (MQ=255)`  
                                                                    `gCCAAAGCGAGCAAACCACGCGACGAAGAAAGCTGGGACGAAGACGACGAAGAGTCCGAGGAAGCAGACGAAGACGGAGACTTCTAAGTGGAACTGCGGGAGAAAATCCTTGAGCGAATCAAGGTGACTTCCTCTGGGTGTTGGGAGTGGCAGGGCGCTACGAACAATAAAGGGTACGGGCAGGTGTGGTGCAGCAATACCGGAAAGGTTGTCTACTGTCATCGCGTAATGTCTAATGCTCCGAAAGGTTCTACCGTCCTGCACTCATGTGATAATCCATTATGTTGTAACCCTGAACAcc                                                                                                                                                                                                                                           >  1:374744/1‑301 (MQ=255)`  
                                                                           `cGAGCAAACCACGCGACGAAGAAAGCTGGGACGAAGACGACGAAGAGTCCGAGGAAGCAGACGAAGACGGAGACTTCTAAGTGGAACTGCGGGAGAAAATCCTTGAGCGAATCAAGGTGACTTCCTCTGGGTGTTGGGAGTGGCAGGGCGCTACGAACAATAAAGGGTACGGGCAGGTGTGGTGCAGCAATACCGGAAAGGTTGTCTACTGTCATCGCGTAATGTCTAATGCTCCGAAAGGTTCTACCGTCCTGCACTCCTGTGATAATCCATTATGTTGTAACACTGAACACCTATcctg                                                                                                                                                                                                                                    >  1:167333/1‑299 (MQ=255)`  
                                                                            `gAGCAAACCACGCGACGAAGAAAGCTGGGACGAAGACGACGAAGAGTCCGAGGAAGCAGACGAAGACGGAGACTTCTAAGTGGAACTGCGGGAGAAAATCCTTGAGCGAATCAAGGTGACTTCCTCTGGGTGTTGGGAGTGGCAGGGCGCTACGAACAATAAAGGGTACGGGCAGGTGTGGTGCAGCAATCCCGGAAAGGTTGTCTACTGTCATCTCGTCATGTCTAATGCTCCGACCGGTTCTACCGTCCTTCACTCCTGTGACAATCCCTTATGTTGTAACACCGCACACCTATCCATa                                                                                                                                                                                                                                   >  1:194240/1‑301 (MQ=255)`  
                                                                              `tgtataagagacaggaagaaAGCTGGGACGAAGACGACGAAGAGTCCGAGGAAGCAGACGAAGACGGAGACTTCTAAGTGGAACTGCGGGAGAAAATCCTTGAGCGAATCAAGGTGACTTCCTCTGGGTGTTGGGAGTGGCAGGGCGCTACGAACAATAAAGGGTACGGGCAGGTGTGGTGCAGCAATACCGGAAAGGTTGTCTACTGTCATCGCGTAATGTCTAATGCTCCGAAAGGTTCTACCGTCCTGCACTCCTGTGATAATCCATTATGTTGTAACCCTGAACACCTATCCATAgg                                                                                                                                                                                                                                 <  1:237162/287‑1 (MQ=255)`  
                                                                              `atgtgtataagagacaggaaAGCGGGGAAGAAGACGACGAAGAGGCAGAGGAAGCAGACGAAGACGGAGACTTCTAAGTGGAACTGCGGGGGAAAATCCTTGAGCGAATCAAGGTGACTTCCTCTGGGTGTTGGGAGTGGCAGGGCGCTACGAACAATAAAGGGTACGGGCAGGTGTGGTGCAGCAATACCGGAAAGGTTGTCTACTGTCATCGCGTAATGTCTAATGCTCCGAAAGGTTCTACCGTCCTGCACTCCTGTGATAATCCATTATGTTGTAACCCTGAACACCTATCCATAgg                                                                                                                                                                                                                                 <  1:100153/284‑1 (MQ=255)`  
                                                                                                      `gggACGAAGACGACGAAGAGTCCGAGGAAGCAGACGAAGACGGAGACTTCTAAGTGGAACTGCGGGAGAAAATCCTTGAGCGAATCAAGGTGACTTCCTCTGGGTGTTGGGAGTGGCAGGGCGCTACGAACAATAAAGGGTACGGGCAGGTGTGGTGCAGCAATACCGGAAAGGTTGTCTACTGTCATCGCGTAATGTCTAATGCTCCGAAAGGTTCTACCGTCCTGCACTCCTGTGATAATCCATTATGTTGTAACCCTGAACACCTATCCATAGGAACTCCAAAAGAGAACTCCACTGa                                                                                                                                                                                                         >  1:265039/1‑301 (MQ=255)`  
                                                                                                        `gagacaGACGATGAAGAGTCCGAGGAAGCAGACGAAGACGGAGACTTCTAAGTGGAACTGCGGGAGAAAATCCTTGAGCGAATCAAGGTGACTTCCTCTGGGTGTTGGGAGTGGCAGGGCGCTACGAACAATAAAGGGTACGGGCAGGTGTGGTGCAGCAATACCGGAAAGGTTGTCTACTGTCATCGCGTAATGTCTAATGCTCCGAAAGGTTCTACCGTCCTGCACTCCTGTGATAATCCATTATGTTGTAACCCTGAACACCTATCCATAGGAACTCCAAAAGAGAACTCCACTGACa                                                                                                                                                                                                       <  1:270655/296‑1 (MQ=255)`  
                                                                                                             `aGACGACGAAGAGTCCGAGGAAGCAGACGAAGACGGAGACTTCTAAGTGGAACTGCGGGAGAAAATCCTTTAGCGAATCAAGGTGACTTCCTCTGGGTGTTGGGAGTGGCAGGGCGCTACGAACAATAAAGGGTACGGGCAGGTGTGGTGCAGCAATACCGGAAAGGTTGTCTACTGTCATCGCGTAATGTCTAATGCTCCGAAAGGTTCTACCGTCCTGCACTCCTGTGATAATCCATTATGTTGTAACCCTGAACACCTATCCATAGGAACTCCAAAAGAGAACTCCACTGACATGGTa                                                                                                                                                                                                  <  1:7937/301‑1 (MQ=255)`  
                                                                                                                 `gacgaAGAGTCCGAGGAAGCAGACGAAGACGGAGACTTCTAAGTGGAACTGCGGGAGAAAATCCTTGAGCGAATCAAGGTGACTTCCTCTGGGTGTTGGGAGTGGCAGGGCGCTACGAACAATAAAGGGTACGGGCAGGTGTGGTGCAGCAATACCGGAAAGGTTGTCTACCGTCATCGCGTAATGTCTAATGCTCCGAAAGGTTCTACCGTCCTGCACTCCTGTGATAATCCATTATGTTGTAACCCTGAACACCTATCCATCGGAACTCCAAAAGAGAACTCCACTGACATGGTAAATa                                                                                                                                                                                              >  1:305066/1‑301 (MQ=255)`  
                                                                                                                  `gaggcaggtacGAGGAAGCAGACGAAGACGGATACTTCTAAGTGGAACGGCGGGAGAAAATCCTTGAGAGAAACAAGGTGACTTCCTCTGGGTGTTGGGAGTGGCAGGGCGCTACGAACAATAAAGGGTACGGGCAGGTGTGGTGCAGCAATACCGGAAAGGTTGTCTACTGTCATCGCGTAATGTCTAATGCTTCGAAAGGTTCTACCGTCCTGCACTCCTGTGATAATCCATTATGTTGTAACCCTGAACACCTATCCATAGGAACTCCAAAAGAGAACTCCACTGACATGGTAAATaa                                                                                                                                                                                             <  1:298806/291‑1 (MQ=255)`  
                                                                                                                  `gacaggagTCCGAGGAAGCAGGCGAAGACGGAGACTTCTAAGTGGAACTGCGGGAGAAAATCCTTGAGCGAATCAAGGTGACTTCCTCTGGGTGGTGGGAGTGGCAGGGCGCTACGAACAATAAAGGGAACGGGCAGGTGTGGTGCAGCAATACCGGTAAAGTTGTCTACTGTCATCGCGTAATGTCTAATGCTCCGAAAGGTTCTACCGTCCTGCACTCCTGTGATAATCCATTATGTTGTAACCCTGAACACCTATCCATAGGAACTCCAAAAGAGAACTCCACTGACATGGTAAATaa                                                                                                                                                                                             <  1:31453/296‑1 (MQ=255)`  
                                                                                                                       `gagTCCGAGGAAGCAGACGAAGACGGAGACTTCTAAGTGGAACTGCGGGAGAAAATCCTTGAGCGAATCAAGGTGACTTCCTCTGGGTGTTGGGAGTGGCAGGGCGCTACGAACAATAAAGGGTACGGGCAGGTGTGGTGCAGCAATACCGGAAAGGTTGTCTACTGTCATCGCGTAATGTCTAATGCTCCGAAAGGTTCTACCGTCCTGCACTCCTGTGATAATCCATTATGTTGTAACCCTGAACACCTATCCATAGGAACTCCAAAAGAGAACTCCACTGACATGGTAAATAAGGGTc                                                                                                                                                                                        >  1:69177/1‑301 (MQ=255)`  
                                                                                                                                    `aaGACGAAGACGGAGACTTCTAAGTGGAACTGCGGGAGAAAATCCTTGAGCGAATCAAGGTGACTTCCGCGTGGTGTTGGGAGTGGCAGGGCGCTACGAACAATAAAGGGTACGGGCAGGTGTGGTGCAGAAATACCGGAAAGGTTGTCTACTGTCATCGCGTAATGTCTAATGCTCCGAAAGGTTCTACCGTCCTGCACTCCTGTGATAATCCTTTATGTTGTAACCCTGAACACCTATCCATAGGAACTCCAAAAGAGAACTCCACTGACATGGTAAATAAGGGTCGCTCACACAAggg                                                                                                                                                                           <  1:378151/300‑1 (MQ=255)`  
                                                                                                                                     `aGACGAAGACGGAGACTTCTAAGGGGAACTGAGGGAGAAAATCCTGGAGCGAATCAAGGTGACTTCCTCTGGGGGTTGGGAGTGGCAGGGCGCTACGAAAAATAAAGGGTATGGGCAGGTGTGGTGCAGCAATACCGGAAAGGTTGTCTACTGTCATCGCGTAATGTCTAATGCTCCGAAAGGTTCTACCGTCCTGCACTCCTGTGATAATCCATTATGTTGTAACCCTGAACACCTATCCATAGGAACTCCAAAAGAGAACTCCACTGACATGGTAAATAAGGGTCGCTCACACAAgggg                                                                                                                                                                          <  1:133459/301‑1 (MQ=255)`  
                                                                                                                                      `gACGAAGACGGAGACTTCTAAGTGGAACTGCGGGAGAAAATCCTTGAGCGAATCAAGGTGACTTCCTCTGGGTGTTGGGAGTGGCAGGGCGCTACGAACAATAAAGGGTACGGGCAGGTGTGGTGCAGCAATACCGGAAAGGTTGTCTACTGTCATCGCGTAATGTCTAATGCTCCGAAAGGTTCTACCGTCCTGCACTCCTGTGATAATCCATTATGTTGTAACCCTGAACACCTATCCATAGGAACTCCAAAAGAGAACTCCACTGACATGGTAAATAAGGGTCGCTCACACAAGGGGt                                                                                                                                                                         <  1:9694/301‑1 (MQ=255)`  
                                                                                                                                      `gACGAAGACGGAGACTTCTAAGTGGAACTGCGGGAGAAAATCCTTGAGCGAATCAAGGTGACTTCCTCTGGGCGTTGGGAGTGGCAGGGCGCTACGAACAATAAAGGGTACGGGCAGGTGTGGTGCAGCAATACCGGCCAGGTTGTCTACTGTCATCGCGTACAGTCTAATGCTCCGAAAGGTTCTACCGTCCTGCACTCATGTGAGAATCCATTCGATTGTAACCATGAACACCTATACATAGGAACTCCAAAAGAGAACTCCACTGACATGGTAAATAAAGGTCGCACACACAAgcggc                                                                                                                                                                         >  1:129490/1‑300 (MQ=255)`  
                                                                                                                                        `cGAAGACGGAGACTTCTAAGTGGAACTGCGGGAGAAAATCCTTGAGCGAATCAAGGTGACTTCATCTGGGTGTTGGGAGTGGCAGGGCGCTACGAACAATAACGGGTACTGGCAGGTGTGGTGCAGCAATACCGGAAAGGTTGTCTACTGTCATCGCGTAATGTCTAATGCTCCGAATGGTTCTACCGTCCTGCACTCCTGTGATAATCCATTATGTTGTAACCCTGAACACCTATCCATATGAACTCCAAAAGAGAACTCCACTGACATGGTAAATAAGGGTCGCTCACACAAGTGGtat                                                                                                                                                                       >  1:345035/1‑301 (MQ=255)`  
                                                                                                                                            `agagacagcTTCTAAGTGGAACGGCGGGAGAAAATCCTTGAGCGAATCAAGGTGACTTCCTCTGGGTGTTGGGAGTGGCAGGGCGCTACGAACAATAAAGGGTACGGGCAGGTGTGGTGCAGCAATACCGGAAAGGTTGTCTACTGTCATCGCGTAATGTCTAATGCTCCGAAAGGTTCTACCGTCCTGCACTCCTGTGATAATCCATTATGTTGTAACCCTGAACACCTATCCATAGGAACTCCAAAAGAGAACTCCACTGACATGGTAAATAAGGGTCGCTCACACAAGGGGTATAAAc                                                                                                                                                                   <  1:475296/293‑1 (MQ=255)`  
                                                                                                                                            `agacaggaCTTCTAAGTGTAACTGCGGGAGAAAAACCTTGAGCTAATCAAGGTGACTTCCTCTGGTTGTTGGGAGTGGCAGGGCGCTACCAACAATAAAGGGTACGGTCAGGTGTGGTGCAGCAATACCTGAAAGGTTGTCTACTGTCATCGCGTAATGTCTAATGCTCCGAAATGTTCTACCGTCCTGCACTCCTGTGATAATCCATTATGTTTTCACCCTGAACACCTATCCATAGGAACTCCAAAAGAGAACTCCACTGACATGGTAAATAAGGGTCGCTCACACAAGGGGTATAAAc                                                                                                                                                                   <  1:201153/295‑1 (MQ=255)`  
                                                                                                                                                    `cTTCTAAGTGGAACTGCGGGAGAAAATCCTTGAGCGAATCAAGGTGACTTCCTCTGGGTGTTGGGAGTGGCAGGGCGCTACGAACAATAAAGGGTACGGTCAGGTGTGGTGCAGCAATACCGGAAAGGTTGTCTACTGTCATCGCGTAATGTCTAATGCTCCGAAAGGTTCTACCGTCCTGCACTCTTGTGATAATCCATTATGTTGTAACCCTGAACACCTATCCATAGGAACTCCAAAAGAGAACTCCACTGACATGGTAACTAAGGGTCGCTCACACAAggggctgtctcttatacac                                                                                                                                                           >  1:103945/1‑286 (MQ=255)`  
                                                                                                                                                              `gtgtataagagacaggtcgttGAGCGAAGCAATGTGACTTCCTCGGGGTGTTGGGAGTAGCAGGGCGCTAAGAACAATAAAGGGTACGGGCAGGTGTGGTGCAGAAATACCGGAAAGGTTGTCTACTGTCATCGCGTAATGTCTAATGCTCCGAAAGGTTCTACCGTCCTGCACTCCTGTGATAATCCATTATGTGGTAACCCTGAACACATATCCATAGGAACTCCAAAAGAGAACTCCACTGACATGGTAAATAAGGGTCGCTCACACAAGGGGTATAAACTTTCAGACGAAGACGTaa                                                                                                                                                 <  1:318309/282‑1 (MQ=255)`  
                                                                                                                                                                 `gacaggggGAAAATCCTTGAGCGAATCAAGGTGACTTCCTCTGGGTGTTGGGAGTGGCAGGGCTCTACGAATAATAAAGGGTACGTGCAGGTGTGGTGCAGCAATACCGGAAATGTTGTCTTCTGTCATCGCGTAATGTCTAATGCTCCGAAAGGTTCTACCGTCCTGCACTCCTGTGATAATCCATTATGTTGTAACCCTGAACACCTATCCATAGGAACTCCAAAAGAGAACTCCGCTGACATGGTAAATAAGGGTCGCTCACACAAGGGGTATAAACTTTCAGACGAAGACGTAATgg                                                                                                                                              <  1:146915/297‑1 (MQ=255)`  
                                                                                                                                                                  `tGCGGGAGAAAATCCTTGAGCGAATCAAGGTGACTTCCTCTGGGTGTTGGGAGTGGCAGGGCGCTACGAACAATAAAGGGTATGGGCAGGTGTGGTGCAGCAATACCGGAAAGGTTGTCTACTGTCATCGCGTAATGTCTAATGCTCCGAAAGGTTCTACCGTCCTGCACTCCTGTGATAATCCATTATGTTGTAACCCTGAACACCTATCCATAGGAACTCCAAAAGAGAACTCCACTGACATGGTAAATAAGGGTCGCTCACACAAGGGGTATAAACTTTCAGACGAAGACGTAATGGc                                                                                                                                             <  1:461258/301‑1 (MQ=255)`  
                                                                                                                                                                             `aTCCTTGAGCGAATCAAGGTGACTTCCTCTTGGTGTTGGGAGTGGCAGGGCGCTACGAACAATAAAGGGTACGGGCAGGTGTGGTGCAGCAATACCGGAAAGGTTGTCTACTGTCATCGCGTAATGTCTAATGCTCCGAAAGGTTCTACCGTCCTGCACTCCTGTGATAATCCATTATGTTGTAACCCTGAACACCTATCCATAGGAACTCCAAAAGAGAACTCCACTGACATGGTAAATAAGGGTCGCTCACACAAGGGGTATAAACTTTCAGACGAATACGTAATGGCAATCATGGAGt                                                                                                                                  >  1:365636/1‑301 (MQ=255)`  
                                                                                                                                                                                            `gtgtataagagacaggggTGTTTGGAGTGGCAGGGCGCTACGAACAATAAAGGGTACGGGCAGGTGTGGTGAAGAAATACCGGAAAGGTTGTCTACTGTCATCGCGTAATGTCTAATGCTCCGAAAGGTTCTACCGTCCTGCACTCCTGTGATAATCCATTATGTTGTAACCCTGAACACCTATCCATAGGAAATCCAAAAGAGAACTCCACTGACATGGTAAATAAGGGTCGCTCACACAAGGGGTATAAACTTTCAGACGAAGACGTAATGGCAATCATGGAGTCCAGCGAGTCCAATg                                                                                                                   <  1:370350/286‑1 (MQ=255)`  
                                                                                                                                                                                                  `aCTTCCTCTGGGTGTTGGGAGTGGCAGGGCGCTACGAACAATAAAGGGTACGGGCAGGTGTTGTGCAGCAATACCGGAAAGGTTGTCTACTGTCATCGCGTAATGTCTAATGCTCCGAAAGGTTCTACCGTCCTGCACTCCTGTGATAATCCATTATGTTGTAACCCTGAACACCTATCCATAGGAACTCCAAAAGAGAACTCCACTGACATGGTAAATAAGGGTCGCTCACACAAGGGGTATAAACTTTCAGACTAAGACGTAATGGCAATCATGGAGTCCAGCGAGTCCAATGTATCCt                                                                                                             <  1:220874/301‑1 (MQ=255)`  
                                                                                                                                                                                                     `agagacaggTGTTGGGGGTGGCAGGGCGCTACGAACAATAAAGGGTACGGGCAGGTGTGGTGCAGCAATACCGGAAAGGTTGTCTACTGTCATCGCGTAATGTCTAATGCTCCGAAAGGTTCTACCGTCCTGCACTCCTGTGATAATCCATTATGTTGTAACCCTGAACACCTATCCATAGGAACTCCAAAAGAGAACTCCACTGACATGGTAAATAATGGTCGCTCACACAAGGGGTATAAACTTTCAGACGAAGACGTAATGGCAATCATGGAGTCCAGCGAGTCCAATGTATCCTTAg                                                                                                          <  1:216611/294‑1 (MQ=255)`  
                                                                                                                                                                                                      `ccTCTTGGTGTTGGGAGTGGCAGGGCGCTACGAACAATAAAGGGTACGGGCAGGTGTGGTGCAGCAATACCGGAAAGGTTGTCTACTGTCATCGCGTAATGTCTAATGCTCCGAAAGGTTCTACCGTCCTGCACTCCTGTGATAATCCATTATGTTGTAACCCTGAACACCTATCCATAGGAACTCCAAAAGAGAACTCCACTGACATGGTAAATAAGGGTCGCTCACACAAGGGGTATAAACTTTCAGACGAAGACGTAATGGCAATCATGGAgctgtctcttatacacatctccgagcc                                                                                                         >  1:242795/1‑275 (MQ=255)`  
                                                                                                                                                                                                           `gggTGTTGGGAGTGGCAGGGCGCTACGAACAATAAAGGGTACGGGCAGGTGTGGTGCAGCAATACCGGAAAGGTTGTCTACTGTCATCGCGTAATGTCTAATGCTCCGAAAGGTTCTACCGTCCTGCACTCCTGTGATAATCCATTATGTTGTAACCCTGAACACCTATCCATAGGAACTCCAAAAGAGAACTCCACTGACATGGTAAATAAGGGTCGCTCACACAAGGGGTATCAACTTTCAGACGAAGACGTAATGGCAATCATGGAGTCCAGCGAGTCCAATGTATCCtctgtctctt                                                                                                    >  1:449575/1‑292 (MQ=255)`  
                                                                                                                                                                                                                 `ggggAGTGGCAGGGCGCTACGAACAATAAAGGGTACGGGCAGGTGTGGTGCAGCAATACCGGAAAGGTTGTCTACTGTCATCGCGTAATGTCTAATGCTCCGAAAGGTTCTACCATCCTGCACTCCTGTGATAATCCATTATGTTGTAACCCTGAACACCTATCCATAGGAACTCCAAAAGAGAACTCCACTGACATGGTAAAT‑AGGGTCGCTCACACAAGGGGTATAAACTTTCAGACGAAGACGTAATGGCAATCATGGAGTCCAGCGAGTCCAATGTATCCTTAGCTCGCACCTATgg                                                                                             <  1:186398/300‑1 (MQ=255)`  
                                                                                                                                                                                                                   `ggAGTGGCAGGGCGCTACGAACAATAAAGGGTACGGGCAGGTGTGGTGCAGCAATACCGGAAAGGTTGTCTACTGTCATCGCGTAATGTCTAATGCTCCGAAAGGTTCTACCGTCCTGCACTCCTGTGATAATCCATTATGTTGTAACCCTGAACACCTATCCATAGGAACTCCAAAAGAGAACTCCACTGACATGGTAAATAAGGGTCGCTCACACAAGGGGTATAAACTTTCAGACGAAGACGTAATGGCAATCATGGAGTCCAGCGAGTCCAATGTATCCTTAGCTCGCACCTATggg                                                                                            >  1:8730/1‑300 (MQ=255)`  
                                                                                                                                                                                                                    `gAGTGGCAGGGCGCTACGAACAATAAAGGGGACGGGCAGGTGTGGTGCAGCAATACCGGAAAGGTTGTCTACTGTCATCGCGTAATGTCTAATGCTCCGAAAGGTTCTACCGTCCTGCACTCCTGTGATAATCCATTATGTTGTAACCCTGAACACCTATCCATAGGAACTCCAAAAGAGAACTCCACTGACATGGTAAATAAGGGTCGCTCACACAAGGGGTATAAACTTTCAGACGAAGACGTAATGGCAATCATGGAGTCCAGCGAGTCCAATGTATCCTTAGCTCGCACCTATGg‑tg                                                                                          <  1:355574/301‑1 (MQ=255)`  
                                                                                                                                                                                                                      `gTGGCAGGGCGCTACGAACAATAAAGGGTACGGGCAGGTGTGGTGCAGCAATACCGGAAAGGTTGTCTACTGTCATCGCGTAATGTCTAATGCTCCGAAAGGTTCTACCGTCCTGCACTCCTGTGATAATCCATTATGTTGTAACCCTGAACACCTATCCATAGGAACTCCAAAAGAGAACTCCACTGACATGGTAAATAAGGGTCGCTCACACAAGGGGTATAAACTTTCAGACGAAGACGTAATGGCAATCATGGAGTCCAGCTAGTCCAATGTATCCTTAGCTCGCACCTATGg‑tttt                                                                                        >  1:285193/1‑298 (MQ=255)`  
                                                                                                                                                                                                                               `ataagagacagaTAAAGGGTACGGGCAGGTGTGGTGCAGCAATACCGGAAAGGTTGTCTACTGTCATCGCGTAATGTCTAATGCTCCGAAAGGTTCTACCGTCCTGCACTCCTGTGATAATCCATTATGTTGTAACCCTGAACACCTATCCATAGGAACTCCAAAAGAGAACTCCACTGACATGGTAAATAAGGGTCGCTCACACAAGGGGTATAAACTTTCAGACGAAGACGTAATGGCAATCATGGAGTCCAGCGAGTCCAATGTATCCTTAGCTCGCACCTATGG‑TGTCTCCCAACAg                                                                               <  1:457139/290‑1 (MQ=255)`  
                                                                                                                                                                                                                                       `gagaggtgtataagagacAGGTGTGGTGCAGCAATACCGGAAAGGTTGTCTACTGTCATCGCGTAATGTCGAATGCTCCGAAAGGTTCTACCGTCCTGCACTCCTGTGATAATTCATTATGTTGTAACCCTGAACACCTATCCATAGGAACTCCAAAAGAGAACTCCACTGACATGGTAAATAAGGGTCGCTCACACAACGGGTATAAACTTTCAGACGAAGACGTAATGGCAATCATGGAGTCCAGCGAGTCCAATGTATCCTTAGCTCGCACCTATGG‑TGTCTCCCAACAGACTATTtg                                                                       <  1:92769/284‑1 (MQ=255)`  
                                                                                                                                                                                                                                        `cAATAAAGGGTACGGGCAGGTGTGGTGTAGCAATACCGGAAAGGTTGTCTACTGTCATCGCGTAATGTCTAATGCTCCGAAAGGTTCTACCGTCCTGCACTCCTGTGATAATCCATTATGTTGTAACCCTGAACACCTATCCATAGGAACTCCAAAAGAGAACTCCACTGACATGGTAAATTAGGGTCGCTCACACAAGGGGTATAAACTTTCAGACGAAGACGTAATGGCAATCATGGAGTCCAGCGAGTCCAATGTATCCTTAGCTCGCACCTATGG‑TGTCTCCCAAcctgtctcttat                                                                      >  1:444485/1‑290 (MQ=255)`  
                                                                                                                                                                                                                                        `cAATAAAGGGTACGGGCAGGTGTGGTGCAGCAATACCGGAAAGGTTGTCTACTGTCATCGCGTAATGTCTAATGCTCCGAAAGGTTCTACCGTCCTGCACTCCTGTGATAATCCATTATGTTGTAACCCTGAACACCTATCCATAGGAACTCCAAAAGAGAACTCCACTGACATGGTAAATAAGGGTCGCTCACACAAGGTGTATAAACTTTCAGACGAAGACGTAATGGCAATCATGGAGTCCAGCGAGTCCAATGTATCCTTAGCTCGCACCTATGG‑TGTCTCCCAAcctgtctgttat                                                                      >  1:504023/1‑290 (MQ=255)`  
                                                                                                                                                                                                                                        `cAATAAAGGGTACGGGCAGGTGTGGTGCAGCAATACCGGAAAGGTTGTCTACTGTCATCGCGTAATGTCTAATGCTCCGAAAGGTTCTACCGTCCTGCACTCCTGTGATAATCCATTATGTTGTAACCCTGAACACCTATCCATAGGAACTCCAAAAGAGAACTCCACTGACATGGTAAATAAGGGTCGCTCACACAAGGGGTATAAACTTTCAGACGAAGACGTAATGGCAATCATGGAGTCCAGCGAGTCCAATGTATCCTTAGCTCGCACCTATGG‑TGtctctgtctcttatacacat                                                                      >  1:336493/1‑285 (MQ=255)`  
                                                                                                                                                                                                                                        `cAATAAAGGGTACGGGCAGGTGTGGTGCAGCAATACCGGAAAGGTTGTCTACTGTCATCGCGTAATGTCTAATGCTCCGAAAGGTTCTACCGTCCTGCACTCCTGTGATAATCCATTATGTTGTAACCCTGAACACCTATCCATAGGAACTCCAAAAGAGAACTCCACTGACATGGTAAATAAGGGTCGCTCACACAAGGGGTATAAACTTTCAGACGAAGACGTAATGGCAATCATGGAGTCCAGCGAGTCCAATGTATCCTTAGCTCGCACCTATGG‑TGTCTCCCAACAGACTATTtgt                                                                      >  1:400717/1‑301 (MQ=255)`  
                                                                                                                                                                                                                                          `aTAAAGGGTACGGGCAGGTGTGGTGCAGCAATACCGGAAAGGTTGTCTACTGTCATCGCGTAATGTCTAATGCTCCGAAAGGTTCTACCGTCCTGCACTCCTGTGATAATCCATTATGTTGTAACCCTGAACACCTATCCATAGGAACTCCAAAAGAGAACTCCACTGACATGGTAAATAAGGGTCGCTCACACAAGGGGTATAAACTTTCAGACGAAGACGTAATGGCAATCATGGAGTCCAGCGAGTCCAATGTATCCTTAGCTCGCACCTATGg‑tgctgtctcttatacacatctccg                                                                    >  1:150504/1‑279 (MQ=255)`  
                                                                                                                                                                                                                                           `aaaaGGGTACGGGAAGGTGTGGTGCAGCAATACCGGAAAGGTGGTCTACTGTCATCGCGTAATGTCTAATGCTCCGAAAGGTTCTACCGTCCTGCACTCCTGTGATAATCCATTATGTGGTAACACTGAACACCTATCAATAGGAACTCCAAAAGATAACACCACTGACATGGTAAATAAGGGGCGCTCACACAAGGGGTATAAACTGTCAGACGAAGACGTAATGGCAATCATGGAGTACAGCGAGTCCAATGTATCCTTAGCTCGCACCTATGG‑AGTCTCCCAACAGACTATTTGTGat                                                                   <  1:74587/300‑1 (MQ=255)`  
                                                                                                                                                                                                                                            `aaaGGGTACGGGCAGGTGTGGTGCAGCAATACCGGAAAGGTTGTCTACTGTCATCGCGTAATGTCTAATGCTCCGAAAGGTTCTACCGTCCTGCACTCCTGTGATAATCCATTATGTTGTAACCCTGAACACCTATCCATAGGAACTCCAAAAGAGAACTCCACTGACATGGTAAATAAGGGTCGCTCACACAAGGGGTATAAACTTTCAGACGAAGACGTAATGGCAATCATGGAGTCCAGCGAGTCCAATGTATCCTTAGCTCGCACCTATGG‑TGTCTCCCAACAGACTATTTGTGata                                                                  <  1:3904/301‑1 (MQ=255)`  
                                                                                                                                                                                                                                            `aaaGGGTACGGGCAGGTGTGGTGCAGCAATACCGGAAAGGTTGTCTACTGTCATCGCGTAATGTCTAATGCTCCGAAAGGTTCTACCGTCCTGCACTCCTGTGATAATCCATTATGTTGTAACCCTGAACACCTATCCATAGGAACTCCAAAAGAGAACTCCACTGACATGGTAAATAAGGGTCGCTCACACAAGGGGTATAAACTTTCAGACGAAGACGTAATGGCAATCATGGAGTCCAGCGAGTCCAATGTATCCTTAGCTCACACCTATGGCTGtctcttatacacatctccgagcc                                                                   >  1:30963/1‑282 (MQ=255)`  
                                                                                                                                                                                                                                                    `cGGGCAGGTGTGGTGCAGCAATACCGGAAAGGTTGTCTACTGTCATCGCGTAATGTCTAATGCTCCGAAAGGTTCTACCGTCCTGCACTCCTGTGATAATCCATTATGTTGTAACCCTGAACACCTATCCATAGGAACTCCAAAAGAGAACTCCACTGACATGGTAAATAAGGGTCGCTCACACAAGGGGTATAAACTTTAAGACGAAGACGTAATGGCAATCATGGAGTCCAGCGAGTCCAATGTATCCTTAGCTCGCCCCTATGG‑TGTCTCCCAAcctgtctcttatacaaaactccga                                                          >  1:326635/1‑278 (MQ=255)`  
                                                                                                                                                                                                                                                     `gggCAGGTGTGGTGCAGCAATACCGGAAAGGTTGTCTACTGTCATCGCGTAATGTCTAATGCTCCGAAAGGTTCTACCGTCCTGCACTCCTGTGATAATCCATTATGTTGTAACCCTGAACACCTATCCATAGGAACTCCAAAAGAGAACTCCACTGACATGGTAAATAAGGGTCGCTCACACAAGGGGTATAAACTTTCAGACGAAGACGTAATGGCAATCATGGAGTCCAGCGAGTCCAATGTATCCTTAGCTCGCACCTATGG‑TGTCTCCCAACAGACTATTTGTGATATACGCAAAg                                                         >  1:112710/1‑301 (MQ=255)`  
                                                                                                                                                                                                                                                     `gggCAGGTGTGGTGCAGCAATACCGGAAAGGTTGTCTACTGTCATCGCGTAATGTCTAATGCTCCGAAAGGTTCTACCGTCCTGCACTCCTGTGATAATCCATTATGTTGTAACCCTGAACACCTATCCATAGGAACTCAAAAAGAGAACTCCACTGACATGGTAAATAAGGGTCGATCACACAAGGGGTATAAACTTTCAGACGAAGACGTAATGGCAATCATGGAGTCCAGCGAGTCCAATGTATCCTTAGCTCGCACCTATGG‑TGTCCCCCAACAGACTATTTGTGATATACGcctgt                                                         >  1:493050/1‑297 (MQ=255)`  
                                                                                                                                                                                                                                                      `ggCAGGTGTGGTGCAGCAATACCGGAAAGGTTGTCTACTGTCATCGCGTAATGTCTAATGCTCCGAAAGGTTCTACCGTCCTGCACTCCTGTGATAATCCATTATGTTGTAACCCTGAACACCTATCCATAGGAACTCCAAAAGAGAACTCCACTGACATGGTAAATAAGGGTCGCTCACACAAGGGGTATAAACTTTCAGACGAAGACGTAATGGCAATCATGGAGTCCAGCGAGTCCAATGTATCCTTAGCTCGCACCTATGG‑TGTCTCCCAACAGACTATTtgtgctgtctcttataa                                                        >  1:12565/1‑288 (MQ=255)`  
                                                                                                                                                                                                                                                      `ggCAGGTGTGGTGCAGCAATACCGGAAAGGTTGTCTACTGTCATCGCGTAATGTCTAATGCTCCGAAAGGTTCTACCGTCCTGCACTCCTGTGATAATCCATTATGTTGTAACCCTGAACACCTATCCATAGGAACTCCAAAAGAGAACTCCACTGACATGGTAAATAAGGGTCGCTCACACAAGGGGTATAAACTTTCAGACGAAGACGTAATGGCAATCATGGAGTCCAGCGAGTCCAATGTATCCTTAGCTCACACCTATGG‑TGTCTCCCAACAGACTATTTGTGATATAcctgtctc                                                        >  1:420285/1‑294 (MQ=255)`  
                                                                                                                                                                                                                                                       `gCAGGTGGGGTGCAGCAATACCGGAAAGGTTTTCTAATGGCATAGCGTAATGTCTAATGCGCCGAAAGGTTCTACCGTCCTGCACTCATGTGATAATCCATTATGTTGTAACCCTGAACAACTATCCATAGGAACTCCAAAAGAGAACTCCACTGACATGGTAAATAAGGGTCGCTCACACAATGGGTATAAACTTTCAGACGAAGACGTAATGGCAATCATGGAGTCCAGCGAGTCCAATGTATCCTTAGCTCGCACCTATGG‑TGTCTCCCAACAGACTATTTGTGATATACGCAAAggg                                                       <  1:107167/301‑1 (MQ=255)`  
                                                                                                                                                                                                                                                       `gCAGATGTGGTGCAGCAATACCGGAAAGGTTGTCTACTGTCATCGCGTAATGTCTAATGCTCCGAAAGGTTCTACCGTCCTGCACTCCTGTGATAATCCATTATGTTGTAACCCTGAACACCTATCCATAGGAACTCCAAAAGAGAACTCCACTGACATGGTAAATAAGGGTCGCTCACACAAGGGGTATAAACTTTCAGACGAAGACGTAATGGCAATCATGGAGTCCAGCGAGTCAAATGTATCCTTAGCTCGCACCTATGG‑TGTCTCCCAACAGACTATTTGTGATATACACAAAggg                                                       <  1:190589/301‑1 (MQ=255)`  
                                                                                                                                                                                                                                                        `cAGGTGTGGTGCAGCAATACCGGAAAGGTTGTCTACTGTCATCGCGTAATGTCTAATGCTCCGAAAGGTTCTACCGTCCTGCACTCCTGTGATAATCCATTATGTTGTAACCCTGAACACCTATCCATAGGAAATCCAAAAGAGAACTCCACTGACATGGTACATAAGGGTAGCTCACACAAGGGGTATAAACTTTCAGACGAAGACGTAATGGCAATAATGGAGTCCAGCGAGTCCAATGTATCCTTAGCTAGCAACTATGG‑TGTCTCCCAACAGACTATTTGTGATATACGCAAAGGGa                                                      >  1:394955/1‑301 (MQ=255)`  
                                                                                                                                                                                                                                                          `ggTGTGGTGCAGCAATACAGGAAAGGTTGTCTACTGTCATCGCGTAATGTCTAATGCTCCGAAAGGTTCTACCGTCCTGCACTCCTGTGATAATCCATTATGTTGTAACACTGAACACCTATCCATAGGAACTCCAAAAGAGAAATCCACTGACATGGTAAATAAGGGTCGCTCACACAAGGGGTATAAACTTTAAGACGAAGACGTAATGGCAATCATGGAGTCCAGCGCGTCCAATGTATCCTTAGCTGGCACCTATGG‑TGTCTCACACCAGACTATTTGTGATGTACGCAAAtggtgg                                                    >  1:292354/1‑301 (MQ=255)`  
                                                                                                                                                                                                                                                           `gtgtGGTGAAGCAATACCGGAAAGGTTGTCTACTGTCATCGCTTAATGTCTAATGCTCCGAAAGGTTCTACCGTCCTGCACTCCTGTGATAATCCATTATGTTGTAACCCTTAACACCTATCCATAGGAACTCCAAAAGAGAACTCCACTGACATGGTAAATAAGGGTCGCTCACACAAGGGGTATAAACTTTCAGACGAAGACGTAATGGCAATCATGGAGTCCAGCGAGTCCAATGTATCCTTAGCTCGCACCTATGG‑TGTCTCCCAACAGACTATTTGTGATATACGCAAAGGGAGGc                                                   <  1:288782/301‑1 (MQ=255)`  
                                                                                                                                                                                                                                                                 `agcagcaATACCGGAAAGGTTGTCTACTGTCATCGCGTAATGTCTAATGCTCCGAAAGGTTCTACCGTCCTGCACTCCTGTGATAATCCATTATGTTGTAACCCTGAACACCTATCCATAGGAACTCCAAAAGAGAACTCCACTGACATGGTAAATAAGGGTCGCTCACACAAGGGGTATAAACTTTCAGACGAAGACGTAATGGCAATCATGGAGTCCAGCGAGTCCAATGTATCCTTAGCTCGCACCTATGG‑TGTCTCCCAACAGACTATTTGTGATATACGCAAAGGGAGGCGACATg                                             <  1:276375/300‑1 (MQ=255)`  
                                                                                                                                                                                                                                                                   `cagcaATACCGGAAAGGTTGTCTACTGTCATCGCGTAATGTCTAATGCTCCGAAAGGTTCTACCGTCCTGCACTCCTGTGATAATCCATTATGTTGTAACCCTGAACACCTATCCATAGGAACTCCAAAAGAGAACTCCACTGACATGGTAAATAAGGGTCGCTCACACAAGGGGTATAAACTTTCAGACGAAGACGTAATGGCAATCATGGAGTCCAGCGAGTCCAATGTATCCTTAGCTCGCACCTATGG‑TGTCTCCCAACAGACTATTTGTGATATACGCAAAGGGAgtagacctgtc                                           >  1:466066/1‑291 (MQ=255)`  
                                                                                                                                                                                                                                                                   `cagcaATACCGGAAAGGTTGTCTACTGTCATCGCGTAATGTCTAATGCTCCGAAAGGTTCTACCGTCCTGCACTCCTGTGATAATCCATTATGTTGTAACCCTGAACACCTATCCATAGGAACTCCAAAAGAGAACTCCAATGACATGGTAAATAAGGGTCGCTCACACAAGGGGTAAAAACTTTCAGACGAAGACGTAATGGCAATCATGGAGTCCAGCGAGTCCAATGTATCCTTAGCTCGCACCGATGG‑TGTCTACCAAACGACTATTTGTGATATACGCAAAGGGAGCCGACATGGc                                           >  1:331269/1‑301 (MQ=255)`  
                                                                                                                                                                                                                                                                        `aTACCGGAAAGGTTGTCTACTGTCATCGCGTAATGTCTAATGCTCCGAAAGGTTCTACCGTCCTGCACTCCTGTGATAATCCATTATGTTGTAACCCTGAACACCTATCCATAGGAACTCCAAAAGAGAACTCCACTGACATGGTAAATAAGGGTCGCTCCCACAAGGGGTATCAACTTTCAGACGAAGACGTACTGGCAATCATGGAGTCCAGCGAGTCCAATGTATCCTTAGCTCGCACCTATGG‑TGTCTCCCAACAGACTATTTGTGATATACGCAAAGGGAGGCGCCATTGCAGGtt                                      >  1:386333/1‑301 (MQ=255)`  
                                                                                                                                                                                                                                                                          `aCCGGAAAGGTTGTCTACTGTCATCGCGTAATGTCTAATGCTCCGAAAGGTTCTACCGTCCTGCACTCCTGTGATAATCCATTATGTTGTAACCCTGAACACCTATCCATAGGAACTCCAAAAGAGAACTCCACTGACATGGTAAATAAGGGTCGCTCACACAAGGGGTATAAACTTTCAGACGAAGACGTAATGGCAATCATGGAGTCCAGCGAGTCCAATGTATCCTTAGCTCGCACCTATGG‑TGTCTCCCAACAGACTATTTGTGATATACGCAAAGGGAGGCGACATGGCAGGTTAc                                    <  1:435509/301‑1 (MQ=255)`  
                                                                                                                                                                                                                                                                            `cGGCAAGGTTGTCTACTGTCATCGCGTAATGTCTAATGCTCCGAAAGGTTCTACCGTCCTGCACTCCTGTGATAATCCATTATGTTGTAACCCTGAACACCTATCCATAGGAACTCCAAAAGAGAACTCCACTGACATGGTAAATAAGGGTCGCTAACACAAGGGGTATAAACTTTCAGACGAAGACGTAATGGCAATCATGGAGTCCAGCGAGTCCAATGTATCCTTAGCTCGCACCTATGG‑TGTCTCCCAACAGCCTATTTGTGATATACGCAAAGGGAGGCGACATGGCAggctgtct                                  >  1:23312/1‑295 (MQ=255)`  
                                                                                                                                                                                                                                                                                   `gTTGTCTACTGTCATCGCGTAATGTCTAATGCTCCGAAAGGTTCTACCGTCCTGCACTCCTGTGATAATCCATTATTTTGTAACCCTTAACACCTATCCATAGGAACTCCAAAATAGAACTCCACTGACATGTTAAATAAGTTTCTCTCACACAAGGTGTATAAACTTTCAGACTAAGACGTATTGGCATTCATGGATTCCAGCTAGTCCAATGTATCCTTAGCTCTCACCTATGG‑TGTCTCTCAACAGACTATTTGTGATATACGCAAAGGGAGGCGATATGGCAGGTTACggatctttt                           >  1:359999/1‑294 (MQ=255)`  
                                                                                                                                                                                                                                                                                             `gTCATCGCGTAATGTCTAATGCTCCGAAAGGTTCTCCCTTCCTGCACTCCTGTGATAATCCATTATGTTGTACCCCTGACCACCTATCCATAGGCACTCCAAAAGAGAACTCCACTGACATGGTAAAAAAGGGTCGCTCACCCAAGGGGTATACACTTTCAGCCGACGACGTAATGGCAATCATGGAGTCCAGCTAGTCCAATGTATCCTTAGCTCGCACCTATGG‑TGTCTCCCAACAGACTCTTTGTGATATACGCAAAGGTAGGCGACCTGCCCGGTTACGCCGCTAAAGGAATCCGaa                 >  1:82705/1‑301 (MQ=255)`  
                                                                                                                                                                                                                                                                                                 `ccgcgTAATGTCTAATGCTACGAAAGGTTCTACCGTCCTGCACTCCTGTGATAATCCATTATGTTGTAACCCTGAACACCTATCCATAGGAACTCCAAAAGAGAACTCCACTGACATGGTAAATAAGGGTCGCTCACACAAGGGGTATAAACTTTCAGACGAAGACGTAAGGGAAATCATGGAGTCCAGCGAGTCCAATGTATCCTTAGCTCGCACCTATGG‑TGTCTCCCAACAGACTATTTGTGATATACGCAAAGGGAGGCGACATGGCAGGTTACGGCGCTAAAGGAATCCGAAAGGt             <  1:452743/300‑1 (MQ=255)`  
                                                                                                                                                                                                                                                                                                    `cgTAATGTCTAATGCTCCGAAAGGTTCTACCGTCCTGCACTCCTGTGATAATCCATTATGTTGTAACCCTGAACACCTATCCATAGGAACTCCAAAAGAGAACTCCACTGACATGGTAAATAAGGGTAGCTCACACAAGGGGTATAAACTTTCAGACGAAGACGTAATGGCAATCATGGAGTCCAGCGAGTCCAATGTATCCTTATCTCGCACCTATGG‑TGTCTCCCAACAGCCTATTTGTGATATACGCAACGGGAGGCGACATGACAGGTTACGGCGCTAACGGAATCCGAAAGGTTgg          >  1:59796/1‑301 (MQ=255)`  
                                                                                                                                                                                                                                                                                                    `cgTAATGTCTAATGCTCCGAAAGGTTCTACCGTCCTGCACTCCTGTGATAATCCATTATGTTGTAAACCTGAACACCTATCCATAGGAACTCCAAAAGAGAACTCCACTGACATGGTAAATAAGGGTCGCTCACACAAGGGGTATAAACTTTCAGACGAAGACGTAATGGCAATCATGGAGTCCAGCGAGTCCAATGTATCCTTAGCTCGCACCTATGG‑TGTCTCCCAACAGACTATTTGTGATATACGCAAAGGGAGGCGACATGGCAGGTTACGGCGCTAAAGGAATCCGAAAGGTTgg          >  1:198077/1‑301 (MQ=255)`  
                                                                                                                                                                                                                                                                                                        `aTGTCTAATGCTCCGAAAGGTTCTACCGTCCTGCACTCCTGTGATAATCCATTATGTTGTAACCCTGAACACCTATCCATAGGAACTCCAAAAGAGAACTCCACTGACATGGTAAATAAGGGTCGCTCACACAAGGGGTATAAACTTTCAGACGAAGACGTAATGGCAATCATGGAGTCCAGCGAGTCCAATGTATCCTTAGCTCGCACCTATGG‑TGTCTCCAAACAGACTATTTGTGATATACGCAAAGGGAGGCGACATGGCCGGTTAAGGCGCTAAAggctgtctcgtatacacatct      >  1:489590/1‑282 (MQ=255)`  
                                                                                                                                                                                                                                                                                                          `gTCTAATGCTCCGAAAGGTTCTACCGTCCTGCACTCCTGTGATAATCCATTATGTTGTAACCCTGAACACCTATCCATAGGAACTCCAAAAGAGAACTCCACTGACATGGTAAATAAGGGTCGCTCACACAAGGGGTATAAACTTTCAGACGAAGACGTAATGGCAATCATGGAGTCCAGCGAGTCCAATGTATCCTTAGCTCGCACCTATGG‑TGTCTCCCAACAGACTATTTGTGATATACGCAAAGGGAGGCGACATGGCAGGTTACGGCGCTAAAGGAATCCGAAAGGTTGGAggtgt    >  1:213028/1‑297 (MQ=255)`  

                                                                                                                                                                                                                                                                                                            `|`                                                                                                                                                                                                                                                                                                               
`CCTTTGGTGGCGGTGAAGACGATTGGGCTGACGAAGTTGAAGAGAACGGCTATGTTGCCTCTGGTTCTGCCAAAGCGAGCAAACCACGCGACGAAGAAAGCTGGGACGAAGACGACGAAGAGTCCGAGGAAGCAGACGAAGACGGAGACTTCTAAGTGGAACTGCGGGAGAAAATCCTTGAGCGAATCAAGGTGACTTCCTCTGGGTGTTGGGAGTGGCAGGGCGCTACGAACAATAAAGGGTACGGGCAGGTGTGGTGCAGCAATACCGGAAAGGTTGTCTACTGTCATCGCGTAATGTCTAATGCTCCGAAAGGTTCTACCGTCCTGCACTCCTGTGATAATCCATTATGTTGTAACCCTGAACACCTATCCATAGGAACTCCAAAAGAGAACTCCACTGACATGGTAAATAAGGGTCGCTCACACAAGGGGTATAAACTTTCAGACGAAGACGTAATGGCAATCATGGAGTCCAGCGAGTCCAATGTATCCTTAGCTCGCACCTATGG‑TGTCTCCCAACAGACTATTTGTGATATACGCAAAGGGAGGCGACATGGCAGGTTACGGCGCTAAAGGAATCCGAAAGGTTGGAGCGTTTC  >  NC_001604.1/9702‑10302`

  

Alignment Legend

Aligned base mismatch/match (shaded by quality score): `ATCG/ATCG` `< 3 ≤` `ATCG/ATCG` `< 10 ≤` `ATCG/ATCG` `< 22 ≤` `ATCG/ATCG` `< 34 ≤` `ATCG/ATCG` `< 38 ≤` `ATCG/ATCG`

Unaligned base: `atcg`    Masked matching base: `atcg`    Alignment gap: `‑`    Deleted base: `‑`

Reads not counted as support for junction

read\_name Not counted due to insufficient overlap past the breakpoint.

read\_name Not counted due to not crossing MOB target site duplication.
